# Supplementary material for: Identification of photoperiod-induced specific miRNAs in the adrenal glands of Sunite sheep (Ovis aries)
Source: Front Vet Sci. 2022 Jul 22;9:888207. doi: 10.3389/fvets.2022.888207 (PMC9354845; doi:10.3389/fvets.2022.888207)
Supplement: Supplementary file 1 [file Data_Sheet_1.ZIP › Supplementary file/miRDeep2 file.zip!/miRDeep2 file/Know_Structure/SP42A1/oar-let-7b.pdf]

miRBase precursor : oar-let-7b
Total read count : 123083
oar-let-7b read count : 122499
remaining reads : 584

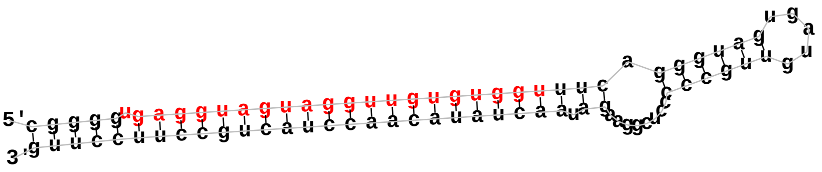

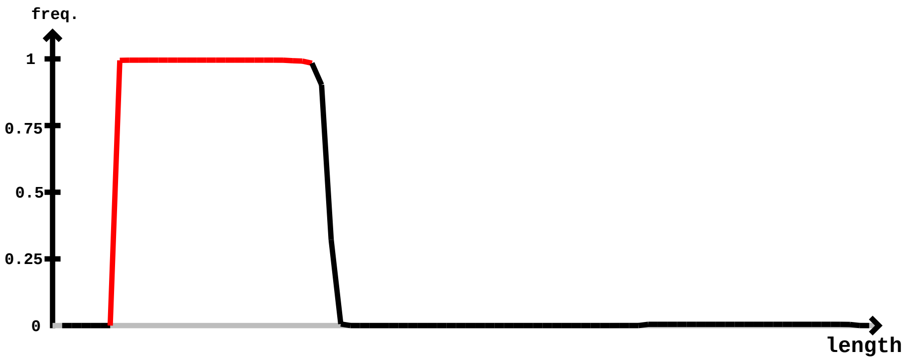


```
       oar-let-7b
5'- cggggugagguaguagguuguguggguuucaggguagugauguugcccccucggaagauaacuauacaaccuacugccuuccuug   -3'    exp
    (((((.(((((((((((((((((((((((.((((((.....))))))(....)..)).))))))))))))))))))))))))))    reads       mm          sample
    .....ugagguaguagguugug..............................................................    17          0           seq
    .....ugagguaguaAguugug..............................................................    1           1           seq
    .....ugagguaguagguugugu.............................................................    244         0           seq
    .....ugagguaguaAguugugu.............................................................    1           1           seq
    .....ugagguaguagguugugug............................................................    177         0           seq
    .....ugagguaguagguugugugg...........................................................    966         0           seq
    .....ugagguaguagguuguguggu..........................................................    9960        0           seq
    .....ugagguaguagguuguguggguu.........................................................    71497       0           seq
    .....ugagguaguagguuguguggguuu........................................................    38811       0           seq
    .....ugagguaguagguuguguggguuuc.......................................................    721         0           seq
    .....ugagguaguagguuguguggguuuca......................................................    5           0           seq
    .....ugagguaguagguuguguggguuucagggu..................................................    1           0           seq
    ......gagguaguagguugugugg...........................................................    1           0           seq
    ......gagguaguagguuguguggu..........................................................    9           0           seq
    ......gagguaguagguuguguggguu.........................................................    54          0           seq
    ......gagguaguagguuguguggguuu........................................................    31          0           seq
    .......agguaguagguuguguggguuu........................................................    2           0           seq
    ........gguaguagguuguguggguu.........................................................    2           0           seq
    ...........................ucaggguagugauguugcccccucggaagaua.........................    2           0           seq
    ......................................auguugcccccucggaagauaa........................    1           0           seq
    ..............................................cccucggaagauaacuauac..................    1           0           seq
    ..................................................cggaagauaacuauacaaccuacu..........    1           0           seq
    ......................................................agauaacuauacaaccuacugccuucc...    1           0           seq
    .........................................................uaacuauacaaccuacugccuuc....    2           0           seq
    .........................................................uaacuauacaaccuacugccuucc...    1           0           seq
    ..........................................................aacuauacaaccuacugccuuccu..    1           0           seq
    ............................................................cuauacaaccuacugccuuc....    4           0           seq
    ............................................................cuauacaaccuacugccuucc...    66          0           seq
    ............................................................cuauacaaccuacugccuuccu..    468         0           seq
    ............................................................cuauacaaccuacugccuuccuu.    29          0           seq
    .............................................................uauacaaccuacugccuucc...    1           0           seq
    .............................................................uauacaaccuacugccuuccu..    5           0           seq
```
